# Kaposi's sarcoma in England and Wales before the AIDS epidemic.

**DOI:** 10.1038/bjc.1992.423

**Published:** 1992-12

**Authors:** A. E. Grulich, V. Beral, A. J. Swerdlow

**Affiliations:** Epidemiological Monitoring Unit, London School of Hygiene and Tropical Medicine, Oxford, UK.

## Abstract

The epidemiological features of Kaposi's Sarcoma (KS) incidence in England and Wales in the period 1971-1980 are reviewed. The epidemiology of KS in England and Wales in this period is distinct from that associated with the AIDS epidemic. The incidence was probably very low compared to other Western countries, there was little male excess, and no indication, based on marital status data, of a raised incidence in male homosexuals. Half the cases registered were in people born outside the UK. The region of birth distribution in these migrants reflected the known pre-AIDS geographic distribution of KS and also pointed to high risks in those from Middle Eastern countries and the Caribbean. The very low incidence rates of KS in natives of England and Wales suggests that the background prevalence of the causative agent for KS was low in England and Wales prior to the AIDS epidemic.


### Kaposi's Sarcoma in England and Wales before the AIDS epidemic

A.E. Grulich[1], V. Beral[2] & A.J. Swerdlow[1]

[1]*Epidemiological Monitoring Unit, London School of Hygiene and Tropical Medicine;* [2]*Imperial Cancer Research Fund Cancer Epidemiology Unit, Oxford, UK.*

**Summary** The epidemiological features of Kaposi's Sarcoma (KS) incidence in England and Wales in the period 1971-1980 are reviewed. The epidemiology of KS in England and Wales in this period is distinct from that associated with the AIDS epidemic. The incidence was probably very low compared to other Western countries, there was little male excess, and no indication, based on marital status data, of a raised incidence in male homosexuals. Half the cases registered were in people born outside the UK. The region of birth distribution in these migrants reflected the known pre-AIDS geographic distribution of KS and also pointed to high risks in those from Middle Eastern countries and the Caribbean. The very low incidence rates of KS in natives of England and Wales suggests that the background prevalence of the causative agent for KS was low in England and Wales prior to the AIDS epidemic.

Before the AIDS epidemic, Kaposi's Sarcoma (KS) was exceedingly rare in Western countries. Anecdotal evidence suggests that the incidence was increased in males of Italian, Eastern European and Jewish ethnic origin (Bluefarb, 1957), but no population-based statistics exist.

In Central and East Africa, KS has long been a common tumour, accounting for more than 10% of all malignancies in males in some countries (Cook *et al.*, 1971). Before AIDS, the sex ratio in Africa was near to 1:1 in children, but in adults over 10 males to each female were affected (Olweny, 1984).

The AIDS-associated epidemic of KS in industrialised countries has epidemiological features which point to a transmissible agent, spread by sexual contact, plus HIV-mediated immunodeficiency, as the likely cause of this neoplasm (Beral *et al.*, 1990). The reported occurrence of KS in homosexual men without HIV infection (Friedman-Kien *et al.*, 1990) raises the possibility that KS may be associated with homosexuality independently of HIV infection. If this is so it might account for the adult male excess of pre-AIDS KS.

In comparison to AIDS-related KS, the epidemiological features of non-AIDS related KS in industrialised countries have been little studied. In this paper we review all incident cases of KS registered in England and Wales during the period 1971-1980 to examine the country of origin, sex ratio and marital status of people with KS before the AIDS epidemic.

### Materials and methods

Data on the 68 cases of KS reported to cancer registries in England and Wales during the period 1971-1980 were obtained from the Office of Population Censuses and Surveys. Age, sex, region of residence, date of registration, date of death (if applicable) and country of birth for these individuals were obtained from cancer registrations. For certain comparisons, similar information was obtained for the 61 cases of KS registered during the period 1981-1985. As KS may be confused diagnostically with haemangiosarcoma (Gottleib *et al.*, 1988), data were also obtained for the 285 cases of haemangiosarcoma registered during the period 1971-1980.

Age- and sex-specific registration rates of KS were calculated using the England and Wales population at the 1981 census as the denominator. Indirectly standardised registration ratios for each region of birth were calculated using the immigrant populations in 1981 as denominators and rates of KS in all individuals with known country of birth as expected rates. Country of birth was unknown for four males and nine females with KS and these were excluded from calculation of standardised registration ratios. Confidence intervals for standardised registration ratios were calculated from tables of multipliers (exact limits) for estimating SMRs (Breslow & Day, 1982).

Marital status is not present in national cancer registration data, but is recorded on death certificates. Thirty people (16 males and 14 females) who were registered with KS in the period 1971-1980, and twenty-seven people (20 males and seven females) who were registered with KS in 1981-85 died before 1986. Data on marital status and cause of death (underlying and contributory) were obtained from extracts of death certificates of these individuals. The proportion of these people who were single in the two time periods was calculated and the difference in these proportions tested for significance using Fisher's exact test. In males, the proportions were adjusted for age differences by applying the England and Wales population 5 year age specific proportions of men who were single at the 1981 census to calculate expected numbers of single men in both time periods. The observed distribution of males who were single between the two time periods was then compared with the expected distribution under a null hypothesis of no difference in age-adjusted marital status between the two time periods, and tested for significance using the binomial distribution.

### Results

Age- and sex-specific registration rates of KS in England and Wales in 1971-1980 are shown in Table I. Rates increased with age in both sexes, and the sex ratio was close to one in each age group. The mean age at registration of KS was 57.1 years in males and 60.2 years in females. In 1981-1985 the mean age at diagnosis in men decreased to 49.3 years, and the sex ratio rose to over two to one, reflecting the onset of the AIDS epidemic. The mean age at diagnosis in women in 1981-1985 remained high, at 69.6 years.

Standardised registration ratios varied enormously according to region of birth (Table II). Significantly raised ratios, of

**Table I** Annual age-specific registration rates per million, and number of registrations of KS, England and Wales, 1971-1980

| | Males | | Females | |
|---|---|---|---|---|
| *Age group* | *Rate* | *n* | *Rate* | *n* |
| 0-14 | 0 | 0 | 0 | 0 |
| 15-39 | 0.08 | 7 | 0.07 | 6 |
| 40-59 | 0.18 | 10 | 0.16 | 9 |
| ⩾ 60 | 0.42 | 17 | 0.33 | 19 |
| All ages | 0.14[a] | 34 | 0.14[a] | 34 |

[a]Age standardised to England and Wales population of 1981.

Correspondence: A.E. Grulich, Department of Epidemiology and Population Sciences, London School of Hygiene and Tropical Medicine, Keppel Street, London WC1E 7HT, UK.
Received 18 December 1991; and in revised form 23 June 1992.

**Table II** Observed and expected numbers of registrations of KS by sex and region of birth, and standardized registration ratios, for all regions of birth with cases of KS, England and Wales, 1971–1980

| *Region of birth* | *Males* Observed | *Males* Expected | *Females* Observed | *Females* Expected | *Both sexes Standardised registration ratio and 95% C.I.* |
|---|---|---|---|---|---|
| England and Wales | 16 | 27.24 | 15 | 22.94 | 0.6 (0.4–0.9) |
| Commonwealth Africa[a] | 4 | 0.10 | 1 | 0.06 | 32.5 (10.6–75.9) |
| North Africa[b] | 0 | 0.02 | 2 | 0.02 | 48.5 (5.9–175.2) |
| Middle East[c] | 2 | 0.03 | 1 | 0.02 | 61.3 (12.6–179.0) |
| Caribbean | 4 | 0.19 | 1 | 0.12 | 16.2 (5.2–37.7) |
| USSR/Poland | 2 | 0.18 | 3 | 0.09 | 19.1 (6.2–44.5) |
| Mediterranean Europe[d] | 1 | 0.19 | 2 | 0.16 | 8.6 (1.8–25.0) |
| Malta and Gozo | 1 | 0.02 | 0 | 0.01 | 32.5 (0.8–181.1) |
| Italy | 0 | 0.06 | 2 | 0.05 | 17.8 (2.2–64.2) |
| Irish Republic | 1 | 0.49 | 0 | 0.39 | 1.1 (0.0–6.1) |

[a]Commonwealth Africa = Kenya, Malawi, Tanzania, Uganda, Zambia, Botswana, Lesotho, Swaziland, Zimbabwe, Gambia, Ghana, Nigeria and Sierra Leone. [b]North Africa = Algeria, Egypt, Libya, Morocco and Tunisia. [c]Middle East = Iran, Israel, Bahrain, Iraq, Jordan, Kuwait, Lebanon, Oman, Qatar, Saudi Arabia, Syria, U.A.E., North Yemen and South Yemen. [d]Mediterranean Europe = Cyprus, Gibraltar, Malta and Gozo, France, Spain, Italy, Greece and Yugoslavia.

30 to 60 times the native England and Wales rates, were found for Commonwealth African, Middle Eastern and North African regions. Eastern European, Mediterranean and Caribbean immigrants also had markedly increased registration ratios. The 13 cases with unknown country of birth were reviewed. Nine had Anglo-Saxon or indeterminate names, two had probably Jewish names, and two had Arabic names.

Amongst the 30 registered in 1971–1980 who died, non-Hodgkin's lymphoma (NHL) was mentioned on the death certificate in three cases. This was 17.6 (95% CI 3.6–51.8) times higher than expected, based on age-specific rates of mention of NHL in multiple cause coding of death certificates in England and Wales (OPCS, 1987). Other malignancies, two of the lung and single mentions of other sites, were recorded on six death certificates, and diabetes mellitus once. Organ transplantation was not mentioned on any death certificate.

None of the 16 men dying whose KS was incident in 1971–1980 were single, whereas eight of 20 males dying whose KS was incident in 1981–1985 were single ($P = 0.005$, Fisher's exact test, 2 tailed). The expected numbers of single men in these time periods calculated from the age-specific porportions of single men at the 1981 census were 1.3 and 2.3 respectively. Thus the expected proportion of all single men dying, who died in the first time period, under the null hypothesis of no difference in marital status between the two time periods, was 0.35 (1.3/3.6), compared to an observed proportion of 0(0/8, $P = 0.03$, binomial distribution). Three of the 14 females dying whose KS was incident before 1981 were single, compared to one of seven dying whose KS was incident between 1981 and 1985 (not significant).

A total of 285 cases of haemangiosarcoma were registered between 1971 and 1980. The mean age at diagnosis was 55.6 years, but it was in general a more fatal tumour than KS, with only 23% of registered cases surviving until 1986, compared to 54% of cases of KS. The male to female ratio was 0.8 to 1. Only 7% (13) of cases of haemangiosarcoma with known country of birth were born overseas, compared to 48% of cases of KS. Eight of the 13 cases of haemangiosarcoma born overseas were from countries identified in this paper as being at high risk of KS.

### Discussion

The registration rates of KS in England and Wales in the 1970's are low. They are about twenty times lower than those reported in the US in the same period (Biggar *et al.*, 1984), although they are about half the rates in a small sample of cities in the US in the 1940s (Oettlé, 1962). The rates are about forty times lower than in Sweden in 1958–1982 (Dictor *et al.*, 1988), but are of a similar order to rates recorded in Denmark before AIDS, which were about one-tenth of those in Sweden (personal communication, M. Melbye). A higher proportion of immigrants from high risk countries might partially account for the high rates in the US, but is very unlikely to account for the high rates in Sweden. Although under-registration of KS needs to be considered as a possible explanation of the low rates in England and Wales, there is no obvious reason why registration of KS should have been much worse than for other cancers (personal communications, Thames and East Anglian cancer registries). Before AIDS, KS was a rare tumour, of interest to dermatologists and pathologists. Its diagnosis required pathological confirmation, which is a source of cancer registrations in the majority of cancer registries. KS can be identified from registration data only by histology coding, but the rates recorded by those cancer registries in England and Wales with high percentages of histologically verified cancers (Muir *et al.*, 1987) are far lower than the rates in the US and Sweden. Finally, the low rates of KS found are consistent with the very few cases of KS we found were recorded at the major specialist skin hospital in England and Wales during this period. It is also possible that some cases of haemangiosarcoma are truly KS, but the very low percentage of cases of haemangiosarcoma born overseas, and the very different survival from the two cancers, suggests that misdiagnosis was not a major problem and could not account for the low rates. Even if every case of haemangiosarcoma registered were truly KS, registration rates would remain lower than rates in the US or Sweden.

KS registration rates in migrants reflect to some extent the geographic distribution of pre-AIDS KS described previously, but the present data appear to be the first population-based statistics which give an indication of the magnitude of the relative risks. Even allowing for the wide confidence intervals, it is clear that migrants from Eastern Europe, Mediterranean countries, Africa, the Middle East and the Caribbean are at an enormously increased risk of KS compared with those born in the UK. Selective migration is unlikely to account for such large variations in relative risk. The high risks in those born in the Middle East and the Caribbean have not been described before, although there have been indirect indications of this from immunosuppressed patients. For example, in Saudi Arabia KS is more common than lymphoma as a complication of renal transplantation (Qunibi *et al.*, 1988), and heterosexuals with AIDS in the US are at increased risk of KS if they were born in the Caribbean (Beral *et al.*, 1990).

The increased incidence of lymphoma seen in subjects with KS has been described previously (Gottleib *et al.*, 1988; Dictor *et al.*, 1988). A much increased incidence of lymphoma has also been reported in immunosuppression (Kinlen, 1982). It is therefore possible that KS may be associated with immune deficiency in the absence of AIDS.

This is also suggested by the increased incidence of KS seen in patients on immunosuppressive therapy (Kinlen, 1982). The absence of post-transplantation KS in our data, although this information may be incomplete because it is based only on death certificates, is consistent with previous findings that KS, although a frequent post-transplantation tumour in some countries, is a rare tumour post transplantation in the United Kingdom (Kinlen, 1982). This rarity of KS even in the presence of medical immunosuppression is compatible with a low background prevalence of the causative agent of KS in England and Wales prior to the AIDS epidemic.

The similar registration rate in males and females is in contrast to reports from other Western countries. In the United States and in Sweden the male to female ratio was about 3 to 1 prior to the AIDS epidemic (Biggar *et al.*, 1984; Dictor *et al.*, 1988). There is no reason to believe that there is sex-specific under-reporting of KS in England and Wales. Given the 10 to 1 sex ratio in Africa before AIDS, it is possible that sex ratios are nearer to 1 in populations with lower incidence of KS.

The high male to female ratio usually described in this disease and the recent report of KS in HIV sero-negative homosexual men (Friedman-Kien *et al.*, 1990) raise the possibility that KS has always been associated with homosexuality. However, the sex ratio of registered cases in England and Wales was close to unity and no male diagnosed during 1971–1980 who died was single. Although marital status is an imperfect indicator of homosexuality and information about marital status was collected only from death certificates, the difference from the 1981–1985 data, in which 40% of males diagnosed with KS in the period who died were single, would seem to indicate that homosexually transmitted infection was not a major cause of KS in England and Wales, a country of low KS incidence, before the AIDS epidemic.

We thank the Office of Population Censuses and Surveys for the provision of data. The Epidemiological Monitoring Unit is funded by the Medical Research Council.

### References

BERAL, V., PETERMAN, T.A., BERKELMAN, R.L. & JAFFE, H.W. (1990). Kaposi's sarcoma among persons with AIDS: a sexually transmitted infection? *Lancet*, **335**, 123–128.

BIGGAR, R.J., HORM, J., FRAUMENI, J.F., GREENE, M.H. & GOEDERT, J.J. (1984). Incidence of Kaposi's sarcoma and mycosis fungoides in the United States including Puerto Rico, 1973–1981. *J.N.C.I.*, **73**, 89–94.

BLUEFARB, S.M. (1957). *Kaposi's Sarcoma: Multiple Idiopathic Hemorrhagic Sarcoma*. Illinois: Charles Thomas.

BRESLOW, N.E. & DAY, N.E. (1982). *Statistical Methods in Cancer Research*, Vol. 2. Lyon: International Agency for Research on Cancer.

COOK, P.J. & BURKITT, D.P. (1971). Cancer in Africa. *Br. Med. Bull.*, **27**, 14–20.

DICTOR, M. & ATTEWELL, R. (1988). Epidemiology of Kaposi's sarcoma in Sweden prior to the Acquired Immuno-Deficiency Syndrome. *Int. J. Cancer*, **42**, 346–351.

FRIEDMAN-KIEN, A.E., SALTZMAN, B.R., CAO, Y., NESTOR, M.S., MIRABILE, M., LI, J.J. & PETERMAN, T.A. (1990). Kaposi's sarcoma in HIV negative homosexual men. *Lancet*, **335**, 168–169.

GOTTLIEB, G. & ACKERMAN, A. (1988). *Kaposi's Sarcoma: A Text and Atlas*. Philadelphia: Lea and Febiger.

KINLEN, L.J. (1982). Immunosuppressive therapy and cancer. *Cancer Surv.*, **1**, 565–583.

MUIR,C., WATERHOUSE, J., MACK, T., POWELL, J., & WHELAN, S. (eds) (1987). *Cancer Incidence in Five Continents, volume V*. Lyon: International Agency for Research on Cancer.

OETTLÉ, A.G. (1962). Geographical and racial differences in the frequency of Kaposi's sarcoma as evidence of environmental or genetic causes. *Symposium on Kaposi's Sarcoma: Unio Internationalis Contra Cancrum*, **18**, 330–363.

OFFICE OF POPULATION CENSUSES AND SURVEYS (1987). *1985 Mortality Statistics, Cause*. Series DH2 no.12, London: HMSO.

OLWENY, C.L.M. (1984). Epidemiology and clinical features of Kaposi's Sarcoma in Tropical Africa. In Friedman-Kien, A. & Laubenstein, L. (eds). *AIDS: the epidemic of Kaposi's Sarcoma and opportunistic infections*. Chicago: Year Book Medical Publishers.

QUNIBI, W., AKHTAR, M., SHETH, K., GINN, H., AL-FURAYH, O., DEVOL, E.B. & TAHER, S. (1988). Kaposi's sarcoma; the most common tumor after renal transplantation in Saudi Arabia. *Am. J. Med.*, **84**, 225–232.


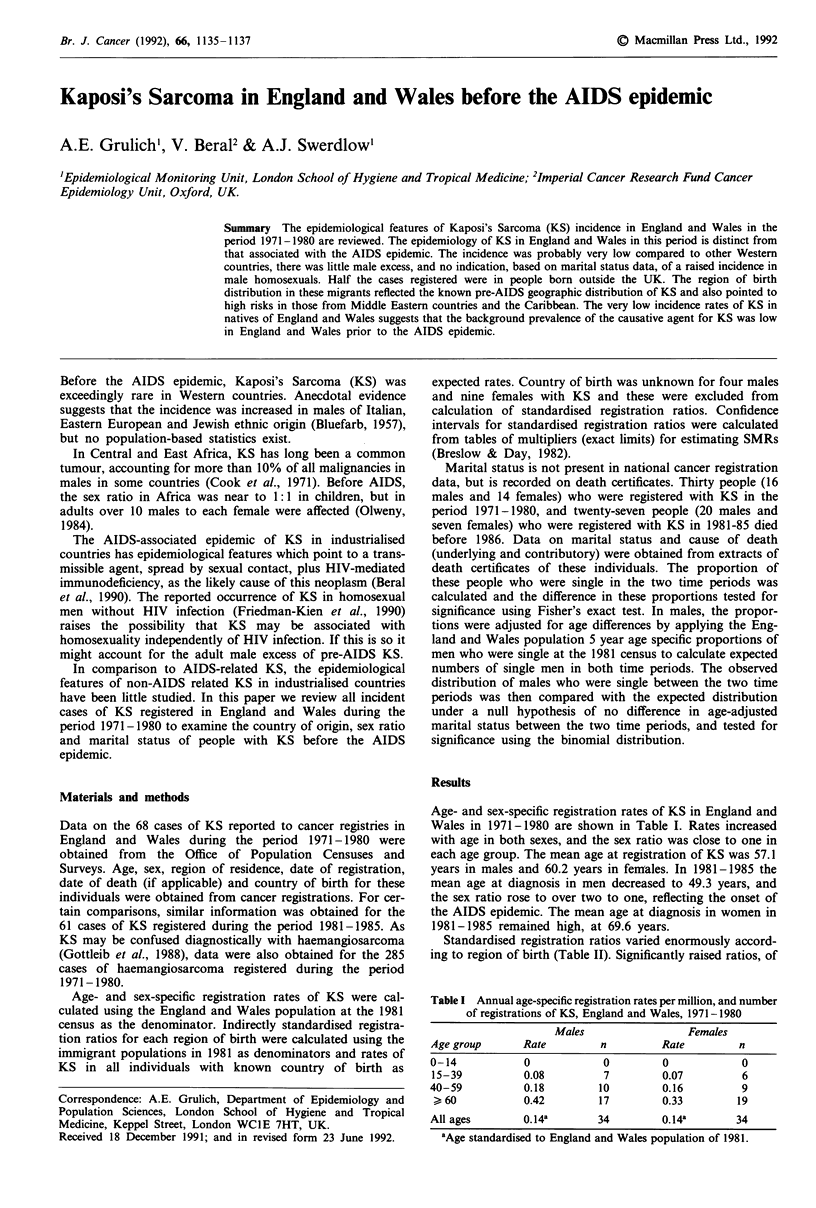

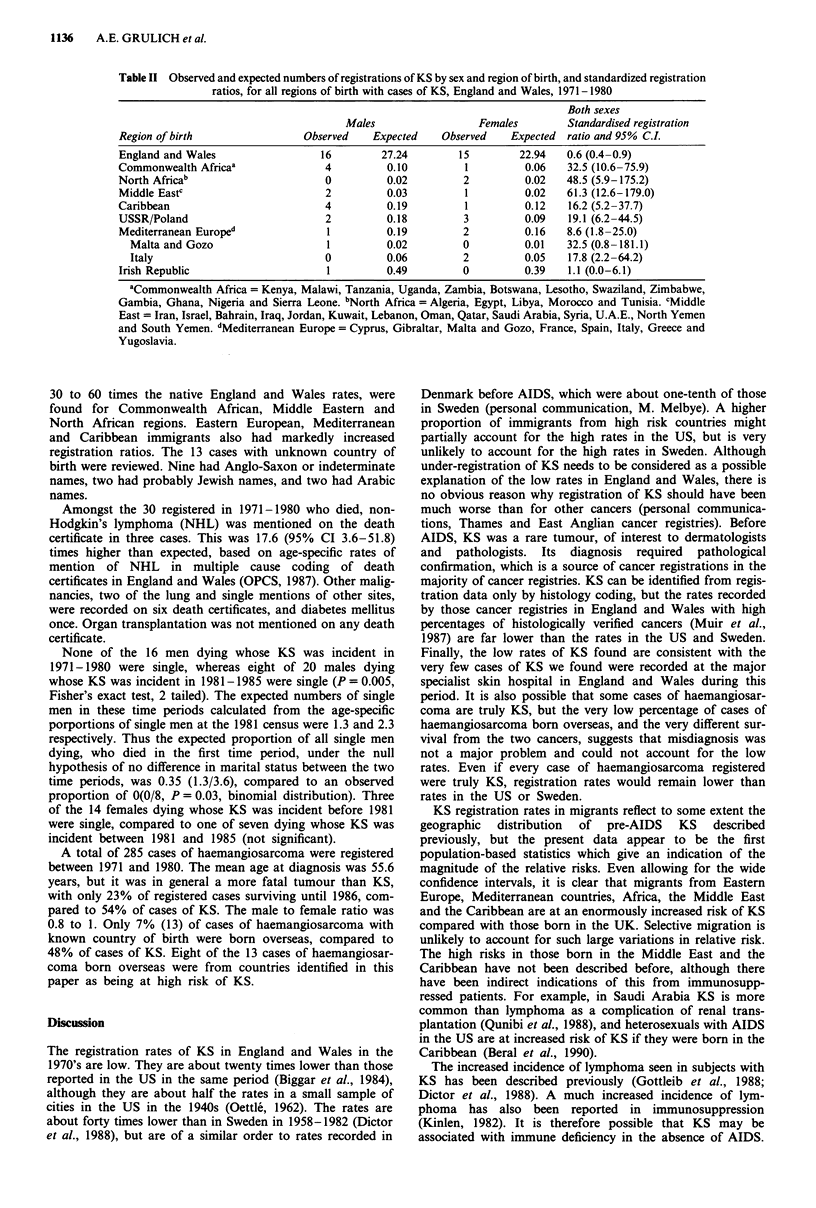

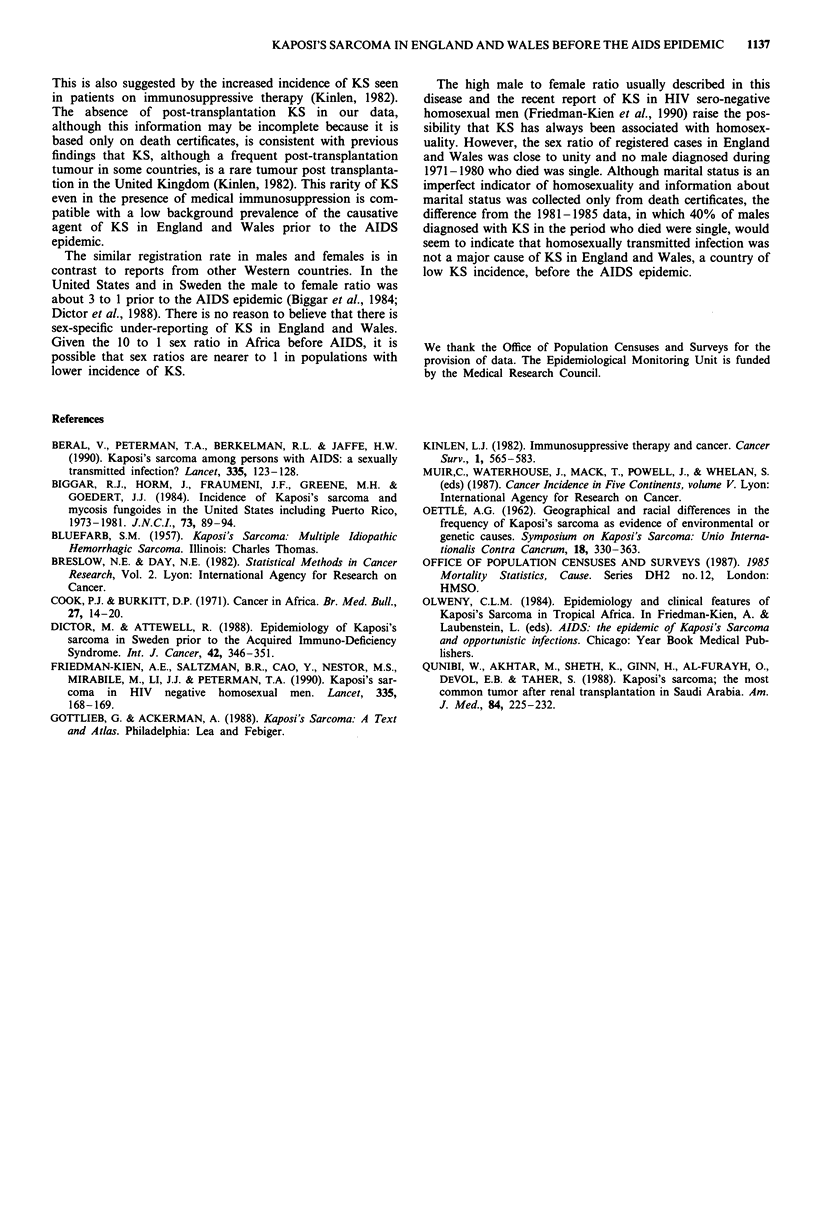


## References

[OCR_00331] Beral V., Peterman T. A., Berkelman R. L., Jaffe H. W. (1990). Kaposi's sarcoma among persons with AIDS: a sexually transmitted infection?. Lancet.

[OCR_00336] Biggar R. J., Horm J., Fraumeni J. F., Greene M. H., Goedert J. J. (1984). Incidence of Kaposi's sarcoma and mycosis fungoides in the United States including Puerto Rico, 1973-81.. J Natl Cancer Inst.

[OCR_00351] Cook P. J., Burkitt D. P. (1971). Cancer in Africa.. Br Med Bull.

[OCR_00355] Dictor M., Attewell R. (1988). Epidemiology of Kaposi's sarcoma in Sweden prior to the acquired immunodeficiency syndrome.. Int J Cancer.

[OCR_00360] Friedman-Kien A. E., Saltzman B. R., Cao Y. Z., Nestor M. S., Mirabile M., Li J. J., Peterman T. A. (1990). Kaposi's sarcoma in HIV-negative homosexual men.. Lancet.

[OCR_00381] OETTLE A. G. (1962). Geographical and racial differences in the frequency of Kaposi's sarcoma as evidence of environmental or genetic causes.. Acta Unio Int Contra Cancrum.

[OCR_00397] Qunibi W., Akhtar M., Sheth K., Ginn H. E., Al-Furayh O., DeVol E. B., Taher S. (1988). Kaposi's sarcoma: the most common tumor after renal transplantation in Saudi Arabia.. Am J Med.

